# Comparison of Medico-Legal Cases Presenting to the Emergency Department Before and After the February 6, 2023 Turkey–Syria Earthquakes

**DOI:** 10.1055/s-0046-1824579

**Published:** 2026-06-26

**Authors:** Oğuzhan Özcan, Erdal Yavuz, Kasim Turgut, Umut Gülaçtı, Irfan Aydın, Ebru Kara

**Affiliations:** 1 Department of Emergency Medicine, Adiyaman University Medical Faculty, Adiyaman, Turkey

**Keywords:** disaster, earthquake, medico-legal case, emergency department

## Abstract

**Background:**

Among natural disasters, earthquakes cause the greatest destruction and are the most devastating. In this study, we examined medico-legal cases presenting to the emergency department and investigated the effect of the earthquake on medico-legal incidents in the society.

**Methods:**

Medico-legal cases that presented to the emergency department of a tertiary level training and research hospital within the 6-month period prior to February 6, 2023 (the date of the earthquakes) and within the 6-month period after the earthquakes were reviewed and compared. Due to the retrospective design of the study, the requirement for informed patient consent was waived.

**Results:**

During the study period, 9,679 medico-legal cases presented to the emergency department, and 8,247 met the inclusion criteria. Of these, 4,978 occurred in the pre-earthquake period and 3,269 in the post-earthquake period. The demographic characteristics differed between periods, with a higher proportion of female patients and a higher mean age in the post-earthquake period. The distribution of medico-legal case types shifted significantly following the disaster. While custody-forensic examinations were the most frequent category prior to the earthquakes, their numbers notably declined afterward, making traffic accidents and assault–battery the predominant cases. Notably, suicide-related poisonings increased among patients without a previous history of psychiatric illness, and there was a marked rise in carbon monoxide exposure and construction-related occupational injuries.

**Conclusion:**

The disaster and its subsequent socio-economic disruptions profoundly altered the profile of medico-legal cases presenting to the emergency department. The observed increase in traffic accidents, construction-related occupational injuries, and atypical poisoning patterns underscore the necessity for customized post-disaster public health interventions and agile resource allocation in emergency care systems.

## Introduction

Disasters are natural or human-made conditions in which large segments of society are affected socially, economically, or physically. Disasters lead to large-scale events that hinder many human activities and create situations in which individuals struggle to cope independently. Among natural disasters, earthquakes are considered the most destructive, uncontrollable, and demographically transformative events with the highest mortality. 1 2 In the clinical context, medico-legal cases encompass any medical presentation that has potential legal implications, including but not limited to traffic accidents, occupational injuries, suspected assaults, poisonings, and custody examinations based on national standardized criteria. 3

Throughout human history, many earthquakes have been recorded in which thousands of people lost their lives and thousands more went missing. 4 Although earthquakes vary in intensity, they may result in destruction-related deaths, injuries (including limb loss), housing problems, loss of loved ones, post-traumatic stress disorder, and severe mental health issues. 5 In Turkey, the February 6, 2023 Turkey–Syria earthquakes were felt severely across 11 provinces, affecting hundreds of thousands of people and resulting in extensive loss of life and property. As in other natural and man-made disasters, individuals who experienced these losses migrated to meet their basic needs such as safety, shelter, food, and water, which led to demographic changes. This event constituted a major catastrophe with international impact, affecting the entire nation. 6

Medico-legal cases may increase during major disasters or population movements affecting society. 7 Post-disaster societies commonly experience migration movements, which alter demographic structures. The fundamental reason for migration is individuals' pursuit of better and safer living conditions. However, it is known that due to the societal effects of these natural or human-made disaster-related migration events, the frequency, distribution, and types of medico-legal cases may change. Furthermore, post-earthquake conditions such as post-traumatic stress disorder, grief, economic difficulties, and urban insecurity are among the causes of medico-legal incidents. 8 9 10

Despite the well-documented immediate casualties of earthquakes, there is a distinct gap in the literature regarding how such large-scale disasters indirectly alter the societal and epidemiological profile of medico-legal emergencies over time. Therefore, this study aimed to evaluate the indirect impact of the February 6, 2023 Turkey–Syria earthquakes on the distribution, mechanism, and characteristics of medico-legal cases presenting to the emergency department, comparing the 6-month periods before and after the event.

## Materials and Methods

### Study Design

This study was a retrospective cohort study involving medico-legal cases that presented to the Emergency Department of Adiyaman Training and Research Hospital between August 6, 2022, and August 6, 2023. During the study period, all medico-legal cases presenting to this hospital were retrospectively examined using the database of a single training and research hospital. The study was initiated after obtaining ethics committee approval from Adıyaman University (decision number: 2023/4-6, dated December 19, 2023). As this was a retrospective study, informed patient consent was not required. Reporting of this study followed the STROBE guidelines.

### Selection of Participants

Adıyaman Training and Research Hospital is the only tertiary hospital in the city center to which all medico-legal cases are referred, including transfers from district hospitals. Crucially, this status as the sole tertiary reference center remained consistent throughout both the pre- and post-earthquake periods. It is the only hospital admitting severe medico-legal cases requiring hospitalization. However, it is important to note that the hospital where the study was conducted sustained partial structural damage due to the earthquakes, which inevitably impacted the operational environment during the post-disaster period. In the hospital data system, all medico-legal patients older than 18 years who presented to the hospital were identified. Patients who met the study criteria and had complete data were included in the study.

### Inclusion Criteria for Cases

1. Presentation to the emergency department.
2. Being older than 18 years.
3. Having a medico-legal case file.

### Exclusion Criteria for Cases

1. Non-medico-legal cases.
2. Applications involving individuals under 18 years of age.
3. Presence of earthquake-related trauma.
4. Incomplete data.

Direct earthquake-related trauma cases were deliberately excluded from the cohort. This crucial methodological step was taken to isolate the disaster's indirect psychosocial, systemic, and environmental impacts on societal behavior, thereby differentiating these secondary medico-legal shifts from the immediate physical casualties directly resulting from structural collapses. Furthermore, cases with incomplete critical data (e.g., missing specific medico-legal categorization or unrecorded emergency department outcomes) were excluded from the final analysis. Specifically, a total of 84 cases—comprising 49 from the pre-earthquake period and 35 from the post-earthquake period—were excluded due to missing records. Although these patients were initially registered as medico-legal cases in the hospital information system, they lacked essential anamnesis records or any clinical documentation detailing the indication for their medico-legal categorization.

**Fig. 1:**
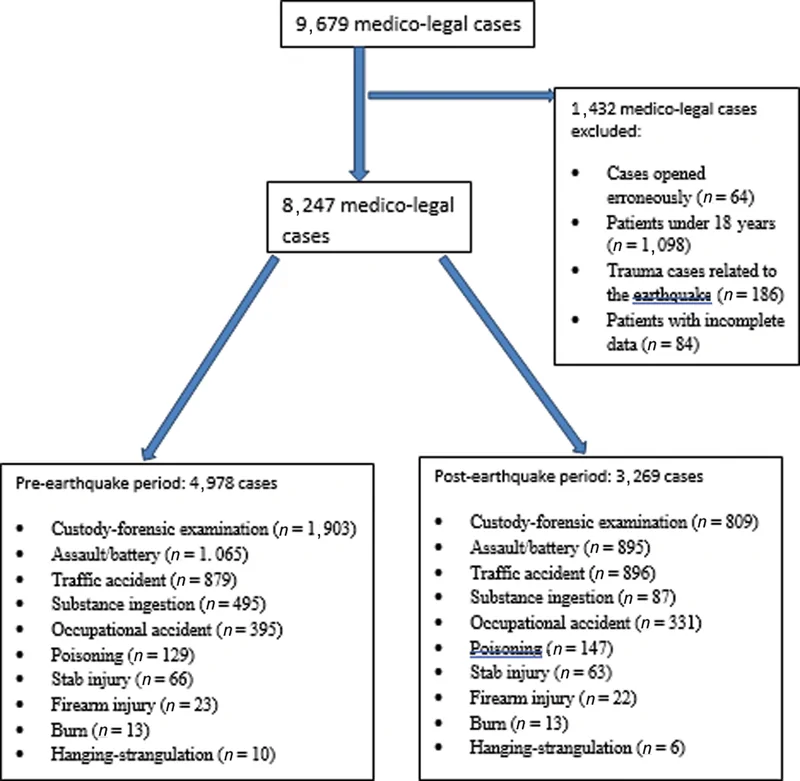
Distribution of medico-legal cases.

After applying the criteria given above, 1,432 patients were excluded for not meeting the requirements. Subsequently, taking February 6, 2023 as the reference date, two groups were formed. There were 4,978 patients in the pre-earthquake period and 3,269 patients in the post-earthquake period. Applications from the two periods were compared in terms of gender, age, type of health insurance, time of presentation, type of medico-legal case, means of arrival, and ward of hospitalization ( Fig. 1 ).

### Study Variables

Demographic characteristics of the patients, such as age and gender, were identified. In the file review of the patients, laboratory data at presentation, imaging methods, discharge summaries, and medico-legal examination reports were examined. These cases were categorized into subgroups according to medico-legal case types. Subsequently, occupational accidents, traffic accidents, and poisonings were evaluated in greater detail. For occupational accidents, the patient discharge summaries and medico-legal reports were examined to detail the injury site, mechanism of occurrence, and emergency department outcomes. Poisoning cases were grouped according to the presence of prior psychiatric illness, the type of agent ingested, the presence of prior suicide attempts, and whether the poisoning was intentional or accidental. Traffic accident cases that resulted in hospital admission after emergency department evaluation were regrouped based on the type of traffic accident, post-admission status, and injury sites. Data were compared between the pre-earthquake and post-earthquake periods.

### Statistical Analysis

Analyses were conducted using the Statistical Package for the Social Sciences (SPSS) v. 17.0 (IBM Corp., NY, USA). Categorical variables were expressed as numbers and percentages. The chi-square test was used to compare categorical data. The normality of the data distribution for continuous variables was assessed using the Kolmogorov-Smirnov test. Continuous variables with a normal distribution were presented as mean ± standard deviation. Comparisons between two groups were analyzed using the Student *t* -test for normally distributed data. *P* values less than 0.05 were considered statistically significant.

## Results

During the study period, a total of 389,071 patients presented to the emergency department. In the pre-earthquake period, there were 212,409 total emergency presentations, from which 4,978 medico-legal cases met the inclusion criteria, constituting 2.34% of all visits. In the post-earthquake period, total emergency presentations decreased to 176,662, with 3,269 medico-legal cases, representing 1.85% of the overall patient volume during this timeframe. The demographic characteristics differed between periods, with a higher proportion of female patients and a higher mean age in the post-earthquake period. Table 1 summarizes the general characteristics of all medico-legal cases, including demographic data, distribution of case types, time of presentation, insurance status, means of arrival, and hospitalized services.

**Table 1:** Comparison of all medico-legal cases between the pre-earthquake and post-earthquake periods

Variables	Pre-earthquake period *n* = 4,978 (%)	Post-earthquake period *n* = 3,269 (%)	*p* -value ^a^
**Gender,** ***n*** **(%)** Female Male	873 (17.5) 4,105 (82.5)	714 (21.8) 2,555 (78.2)	<0.001
**Age (years)**	33.23 ± 12.92	35.48 ± 13.5	<0.001 b
**Type of medico-legal case,** ***n*** **(%)** Burn Traffic accident Occupational accident Poisoning Assault–battery Substance analysis Custody-forensic Stab injury Firearm injury Hanging-strangulation	13 (0.3) 879 (17.7) 395 (7.9) 129 (2.6) 1,065 (21.4) 495 (9.9) 1,903 (38.2) 66 (1.3) 23 (0.5) 10 (0.2)	13 (0.4) 896 (27.4) 331 (10.1) 147 (4.5) 895 (27.4) 87 (2.7) 809 (24.7) 63 (1.9) 22 (0.7) 6 (0.2)	<0.001
**Time of presentation,** ***n*** **(%)** 08:00–16:00 16:00–24:00 24:00–08:00	1,673 (33.6) 2,143 (43) 1,162 (23.3)	1,386 (42.4) 1,440 (44.1) 443 (13.6)	<0.001
**Health insurance/Institution type,** ***n*** **(%)** Forensic case SSI Retirement fund Voluntary insurance Syrian Detainee Green card Other	3,761 (75.6) 107 (2.1) 36 (0.8) 69 (1.4) 50 (1) 835 (16.8) 31 (0.6) 89 (1.8)	2,305 (70.5) 72 (2.2) 38 (1,2) 50 (1.5) 44 (1.3) 694 (21.2) 23 (0.7) 43 (1.3)	<0.001
**Means of arrival,** ***n*** **(%)** Ambulance Own means	535 (10.7) 4,443 (89.3)	336 (10.3) 2,933 (89.7)	0.498
**Hospitalized service,** ***n*** **(%)** Urology Neurosurgery General surgery Thoracic surgery Ophthalmology Cardiovascular surgery Otorhinolaryngology Orthopedics Plastic surgery Intensive care Internal medicine Psychiatry	2 (0.6) 53 (14.8) 24 (6.7) 12 (3.4) 3 (0.8) 6 (1.7) 0 (0) 63 (17.6) 24 (6.7) 150 (42) 16 (4.5) 4 (1.1)	2 (1.1) 24 (12.9) 6 (3.2) 22 (11.8) 1 (0.5) 2 (1.1) 4 (2.2) 37 (19.9) 5 (2.7) 55 (29.6) 26 (14) 2 (1.1)	<0.001

Abbreviation: SSI, social security institution.

Notes: ^a^ Chi-square test.

b Student *t* -test.

The distribution of medico-legal case types shifted significantly after the earthquakes. During the pre-earthquake period, the most prevalent medico-legal cases, in descending order of frequency, were custodial forensic examinations (38.2%), assault and battery (21.4%), and traffic accidents (17.7%). In the post-earthquake period, this distribution shifted to traffic accidents (27.8%), assault and battery (27.4%), custodial forensic examinations (24.7%), and occupational accidents (10.1%). The variation observed in the distribution of medico-legal case types between the two periods was statistically significant ( *p* < 0.001) ( Table 1 ).

Furthermore, hospital admission patterns exhibited significant alterations between the periods. Prior to the earthquake, the distribution of hospital admissions (the top three departments) was as follows: the intensive care unit (ICU) (42%), orthopedics (17.6%), and neurosurgery (14.8%). Following the earthquake, this distribution transitioned to the ICU (29.6%), orthopedics (19.9%), and internal medicine (14%). The difference regarding the clinical departments to which medico-legal cases were admitted was statistically significant ( *p* < 0.001). Additionally, both the time of presentation and the type of health insurance coverage demonstrated significant differences in the post-earthquake period ( *p* < 0.001) ( Table 1 ).

Among the medico-legal cases, a total of 291 patients presented with poisoning: 144 during the pre-earthquake period and 147 during the post-earthquake period. The gender distribution did not differ significantly between the pre-earthquake (58.1% female, 41.9% male) and post-earthquake (55.1% female, 44.9% male) periods ( *p* = 0.624). However, the mean age significantly increased from 32.51 ± 13.549 years in the pre-earthquake period to 34.61 ± 14.565 years in the post-earthquake period ( *p* < 0.001). Furthermore, the proportion of poisoning cases among patients without a prior psychiatric diagnosis was significantly higher following the earthquake ( *p* < 0.001). Regarding a history of prior suicide attempts, no statistically significant difference was observed between the pre-earthquake (11.3%) and post-earthquake (7.5%) cohorts ( *p* = 0.281) ( Table 2 ).

**Table 2:** Comparison of poisoning cases

Variables	Pre-earthquake period, *n* = 144 (%)	Post-earthquake period *n* = 147 (%)	*p* -value ^a^
**Gender,** ***n*** **(%)** Female Male	72 (58.1) 52 (41.9)	81 (55.1) 66 (44.9)	0.624
**Age (years)**	32.51 ± 13.549	34.61 ± 14.565	<0.001 b
**Time of presentation,** ***n*** **(%)** 08:00–16:00 16:00–24:00 24:00–08:00	35 (28.2) 54 (43.5) 35 (28.2)	36 (24.5) 45 (30.6) 66 (44.9)	0.015
**Means of arrival,** ***n*** **(%)** Ambulance Own means	21 (16.9) 103 (83.1)	18 (12.2) 129 (87.8)	0.273
**Hospitalized service,** ***n*** **(%)** Intensive care Ward	3 (12) 22 (88)	0 (0) 32 (100)	0.044
**Psychiatric diagnosis,** ***n*** **(%)** Present Absent	43 (34.7) 81 (65.3)	18 (12.2) 129 (87.8)	<0.001
**Previous suicide attempt,** ***n*** **(%)** Present Absent	14 (11.3) 110 (88.7)	11 (7.5) 130 (92.5)	0.281
**Combined drug ingestion,** ***n*** **(%)** Present Absent	21 (16.9) 103 (83.1)	11 (7.5) 136 (92.5)	0.016
**Analgesic ingestion,** ***n*** **(%)** Present Absent	30 (24.2) 94 (75.8)	20 (13.6) 127 (86.4)	0.025
**Antibiotic ingestion,** ***n*** **(%)** Present Absent	9 (7.3) 115 (92.7)	6 (4.1) 141 (95.9)	0.255
**Substance use,** ***n*** **(%)** Present Absent	0 (0) 124 (100)	19 (12.9) 128 (87.1)	<0.001
**Corrosive substance ingestion,** ***n*** **(%)** Present Absent	7 (5.6) 117 (94.4)	5 (3.4) 142 (96.6)	0.371
**Psychiatric drug ingestion,** ***n*** **(%)** Present Absent	21 (16.9) 103 (83.1)	13 (8.8) 134 (91.2)	0.045
**Internal medicine drug ingestion,** ***n*** **(%)** Present Absent	21 (16.9) 103 (83.1)	19 (12.9) 128 (87.1)	0.354
**Pesticide ingestion,** ***n*** **(%)** Present Absent	5 (4) 119 (96)	4 (2.7) 143 (97.3)	0.548
**Carbon monoxide exposure,** ***n*** **(%)** Present Absent	20 (16.1) 104 (83.9)	73 (49.7) 74 (50.3)	<0.001
**Other,** ***n*** **(%)** Present Absent	37 (29.8) 97 (70.2)	1 (0.7) 146 (99.3)	<0.001

Notes: ^a^ Chi-square test.

b Student *t* -test.

Significant changes were also noted in patterns of substance ingestion, including a decrease in analgesic and combined drug ingestion and a marked increase in carbon monoxide exposure and illicit substance use. Table 2 presents a comparison of poisoning cases, including demographic features, clinical characteristics, psychiatric history, and patterns of agent.

An analysis of poisoning cases revealed no incidents involving illicit substances during the pre-earthquake period; however, in the post-earthquake period, 12.9% of these patients attempted suicide using illicit substances. This difference was statistically significant ( *p* < 0.001). Furthermore, the incidence of carbon monoxide (CO) exposure within this group increased significantly, rising from 16.1% in the pre-earthquake period to 49.7% following the earthquake ( *p* < 0.001).

During the study period, a total of 218 patients were hospitalized due to traffic accidents (128 pre-earthquake and 90 post-earthquake). A significant demographic shift in gender distribution was observed among these patients: the proportion of males increased from 74.8% (25.2% female) in the pre-earthquake period to 87.8% (12.2% female) in the post-earthquake period ( *p* = 0.018). Additionally, the mean age of patients hospitalized for traffic accidents increased significantly from 36.45 ± 18.942 years to 39.92 ± 17.332 years post-earthquake ( *p* < 0.001) ( Table 3 ).

**Table 3:** Comparison of hospitalized traffic accident cases

Variables	Pre-earthquake period, *n* = 127 (%)	Post-earthquake period, *n* = 90 (%)	*p* -value ^a^
**Gender,** ***n*** **(%)** Male Female	95 (74.8) 32 (25.2)	79 (87.8) 11 (12.2)	0.018
**Age (years)**	36.45 ± 18.942	39.92 ± 17.332	<0.001 b
**Time of presentation,** ***n*** **(%)** 08:00–16:00 16:00–24:00 24:00–08:00	48 (37.8) 66 (52) 13 (10.2)	34 (37.8) 47 (52.2) 9 (10)	0.998
**Means of arrival,** ***n*** **(%)** Ambulance Own means	38 (29.9) 89 (70.1)	27 (30) 63 (70)	0.990
**Type of accident,** ***n*** **(%)** Motor vehicle occupant Motor vehicle non-occupant Motorcycle	40 (31.5) 31 (24.4) 56 (44.1)	30 (33.3) 22 (24.4) 38 (42.2)	0.952
**Hospitalized service,** ***n*** **(%)** Urology Neurosurgery General surgery Thoracic surgery Ophthalmology Otorhinolaryngology Orthopedics Plastic surgery Intensive care	1 (0.8) 33 (26) 11 (8.7) 5 (3.9) 1 (0.8) 0 (0) 43 (33.9) 5 (3.9) 28 (22)	2 (2.2) 18 (20) 3 (3.3) 17 (18.9) 0 (0) 2 (2.2) 22 (26.7) 2 (2.2) 24 (26.7)	0.006
**Cranial trauma,** ***n*** **(%)** Present Absent	49 (38.6) 78 (61.4)	36 (40) 54 (60)	0.833
**Spinal trauma,** ***n*** **(%)** Present Absent	16 (12.6) 111 (87.4)	12 (13.3) 78 (86.7)	0.874
**Thoracic trauma,** ***n*** **(%)** Present Absent	18 (14.2) 109 (85.8)	30 (33.3) 60 (66.7)	0.001
**Abdominal trauma,** ***n*** **(%)** Present Absent	12 (9.4) 115 (90.6)	10 (11.1) 80 (88.9)	0.689
**Extremity trauma,** ***n*** **(%)** Present Absent	59 (46.5) 68 (53.5)	40 (44.4) 50 (55.5)	0.769
**Other traumas,** ***n*** **(%)** Present Absent	1 (0.8) 126 (99.2)	0 (0) 90 (0)	0.585
**Post-hospitalization outcome,** ***n*** **(%)** Discharge Death Referred	123 (96.9) 4 (3.1) 0 (0)	84 (93.3) 0 (0) 6 (6.7)	0.003

Notes: ^a^ Chi-square test.

b Student *t* -test.

Regarding the anatomical distribution of injuries in the pre-earthquake period, patients presented with cranial (38.6%), spinal (12.6%), thoracic (14.2%), abdominal (9.4%), and extremity (46.5%) trauma, alongside other localized injuries (e.g., ear, nose, or eye trauma) (0.8%). In the post-earthquake period, the distribution shifted to cranial (40%), spinal (13.3%), thoracic (33.3%), abdominal (11.1%), and extremity (44.4%) trauma. A comparative analysis of trauma types revealed that only the incidence of thoracic trauma exhibited a statistically significant increase in the post-earthquake period ( *p* = 0.001) ( Table 3 ). Table 3 provides detailed comparisons of hospitalized traffic-accident cases, including demographic characteristics, accident type, trauma regions, hospital services, and outcomes.

Among the medico-legal cases, a total of 726 patients presented to the emergency department due to occupational accidents during the study period (395 in the pre-earthquake period and 331 in the post-earthquake period). Occupational accidents predominantly involved males across both periods. Specifically, males accounted for 91.6% (8.4% females) of the cases in the pre-earthquake period and 89.7% (10.3% females) in the post-earthquake period; this gender distribution did not exhibit a statistically significant difference ( *p* = 0.790). However, the mean age of the patients significantly increased from 36.45 ± 18.942 years in the pre-earthquake period to 39.92 ± 17.332 years in the post-earthquake period ( *p* < 0.001) ( Table 4 ).

**Table 4:** Comparison of occupational accident cases

Variables	Pre-earthquake period *n* = 395 (%)	Post-earthquake period *n* = 331 (%)	*p* -value ^a^
**Gender,** ***n*** **(%)** Male Female	362 (91.6) 33 (8.4)	297 (89.7) 34 (10.3)	0.790
**Age (years)**	35.74 ± 12.036	34.50 ± 11.258	<0.001 b
**Time of presentation,** ***n*** **(%)** 08:00–16:00 16:00–24:00 24:00–08:00	234 (59.2) 133 (33.7) 28 (7.1)	208 (62.8) 103 (31.1) 20 (6)	0.594
**Means of arrival,** ***n*** **(%)** Ambulance Own means	52 (13.2) 343 (86.8)	22 (6.6) 309 (93.4)	0.004
**Hospitalized service,** ***n*** **(%)** Neurosurgery General surgery Thoracic surgery Ophthalmology Cardiovascular surgery Otorhinolaryngology Orthopedics Plastic surgery Intensive care	4 (8) 0 (0) 0 (0) 0 (0) 2 (4) 0 (0) 20 (40) 10 (20) 14 (28)	3 (7.9) 3 (7.9) 1 (2.6) 2 (5.3) 1 (2.6) 1 (2.6) 19 (50) 0 (0) 8 (21.1)	0.023
**Sector of employment (%)** Public sector employee Factory Small business Construction Agricultural worker	22 (5.6) 170 (43) 49 (12.4) 150 (38) 4 (1)	38 (11.5) 110 (33.2) 42 (12.7) 139 (42) 2 (0.6)	0.01
**Emergency outcome,** ***n*** **(%)** Death Discharge Admission	0 (0) 345 (87.3) 50 (12.7)	2 (0.9) 293 (88.5) 36 (10.9)	0.235
**Type of injury,** ***n*** **(%)** Penetrating Blunt	126 (31.9) 269 (68.1)	107 (32.3) 224 (67.7)	0.430
**Cranial trauma,** ***n*** **(%)** Present Absent	65 (16.5) 330 (83.5)	69 (20.8) 262 (79.2)	0.129
**Spinal trauma,** ***n*** **(%)** Present Absent	24 (6.1) 371 (93.9)	25 (7.6) 306 (92.4)	0.430
**Thoracic trauma,** ***n*** **(%)** Present Absent	13 (3.3) 382(96.7)	19 (5.7) 312(94.3)	0.109
**Abdominal trauma,** ***n*** **(%)** Present Absent	6(1.5) 389(98.5)	9(2.7) 322(97.3)	0.258
**Extremity trauma,** ***n*** **(%)** Present Absent	279 (70.6) 116 (29.4)	218 (65.9) 113 (34.1)	0.168
**Ophthalmological injury,** ***n*** **(%)** Present Absent	22 (5.6) 373 (94.4)	6 (1.8) 325 (98.2)	0.009
**Other traumas,** ***n*** **(%)** Present Absent	18 (4.6) 377 (95.4)	18 (5.4) 313 (94.6)	0.353

Notes: ^a^ Chi-square test.

b Student *t* -test.

Regarding the distribution by employment sector, pre-earthquake patients were primarily factory workers (42%), followed by those in the construction sector (38%), small businesses (12.4%), public employment (5.6%), and agriculture (1%). In contrast, during the post-earthquake period, the construction sector accounted for the highest proportion of cases (42%), followed by factory workers (33.2%), small business employees (12.7%), public employees (11.5%), and agricultural workers (2%). The variation observed among employment sectors between the two periods was statistically significant ( *p* = 0.010) ( Table 4 ). Table 4 summarizes the comparison of occupational accident cases, including demographic data, work sectors, hospital admissions, and injury mechanisms.

## Discussion

This study highlights a significant shift in the epidemiological profile of medico-legal cases following the February 6, 2023 Turkey–Syria earthquakes. While the overall volume of custody-forensic examinations decreased, there was a pronounced increase in traffic accidents, construction-related occupational injuries, carbon monoxide exposures, and suicide-related poisonings among individuals without a prior psychiatric history.

Medico-legal incidents are generally seen more frequently in men. The reason for the predominance of male cases is that men participate in social life more than women and spend more time outside compared to women. 3 Our study is similar to other studies in this regard. In previous studies, the mean age of medico-legal cases ranged between 20 and 40 years. 3 11 In our study, the mean age was 33.23 years for the pre-earthquake period and 35.48 years for the post-earthquake period, consistent with the literature.

In the evaluation performed in terms of medico-legal case type, the most common medico-legal case type in the pre-earthquake period was custody-forensic examination, whereas in the post-earthquake period, traffic accidents and assault–battery cases were predominant. In a study by Zeren et al, 12 traffic accidents constituted the most common medico-legal case group, and similar results were observed in another study by Turla et al, 13 where traffic accidents ranked first among medico-legal cases. However, in our study, custody-forensic examinations were the most common type of presentation in the pre-earthquake period, contrary to the literature. In the period after the disaster, traffic accidents, assault–battery, and occupational accidents increased.

Among poisoning cases, 34.7% of patients in the pre-earthquake period had a history of psychiatric illness, whereas in the post-earthquake period, the proportion of patients with a psychiatric diagnosis decreased to 12.2%. In a study by Yavuz and Turgut, 14 a history of psychiatric illness was found to significantly increase the risk of suicide attempts. Another study showed that most patients who attempted suicide had recently received psychiatric treatment. 15 16 17 In our study, similar to the literature, poisoning cases were more frequent among psychiatric patients in the pre-earthquake period. However, the presence of a prior psychiatric diagnosis among poisoning cases decreased significantly in the post-earthquake period. We hypothesize that the emergence of suicidal tendencies in previously undiagnosed individuals is driven by the profound sense of helplessness, acute grief, and severe socio-economic disruption following the disaster. Such catastrophic events can trigger acute stress responses and suicidal inclinations even in individuals without documented prior psychiatric vulnerabilities. 4 8

Ahmad et al 18 stated that substance misuse may increase after major disasters and traumas. In our study, no poisoning presentations due to substance use were recorded in the pre-earthquake period, whereas in the post-earthquake period, 12.9% of poisoning cases were due to substance use, suggesting an increase in maladaptive coping mechanisms in response to severe psychological trauma. 18 Furthermore, the marked increase in carbon monoxide exposure post-earthquake is a critical disaster-related finding. This surge is likely attributable to the widespread disruption of electrical infrastructure, forcing survivors to rely on unsafe heating methods, such as unventilated stoves or fuel-powered generators inside temporary shelters.

Upon examining traffic accidents in terms of accident type, we observed that in the pre-earthquake period, 31.5% were motor vehicle occupant (MVO) accidents, 24.4% were motor vehicle non-occupant (MVNO) accidents, and 44.1% were motorcycle accidents. In the post-earthquake period, 33.3% were MVO accidents, 24.4% were MVNO accidents, and 42.2% were motorcycle accidents. In a study by Aydeniz et al, 19 MVO accidents constituted 58.6%, MVNO accidents 30.1%, and motorcycle accidents 10.7%. In a study by Aktaş et al, 20 the rate of MVO accidents was 62%, MVNO accidents 21%, and motorcycle accidents 12%. In another study by Küçüker and Aksu, 21 41.8% were MVO accidents, 41.2% MVNO accidents, and 17% motorcycle accidents. The comparison between the two periods in our study was not statistically significant. The proportion of motorcycle accidents in our study was found to be higher than the values reported in the literature.

When the patients who were involved in a traffic accident were grouped according to the wards to which they were admitted, orthopedic clinics had the highest admission rates in both periods. Hospital admissions to the ICU and thoracic surgery clinic increased in the post-earthquake period compared to the pre-earthquake period. In a study by Varol et al, 22 the highest hospitalization rate among traffic accident patients presenting to the emergency department was in orthopedics (24.2%), followed by neurosurgery (12.6%). In a study by Aktaş et al, 23 among medico-legal trauma cases presenting to the emergency department, the highest hospitalization rate was again in orthopedics (24.2%), followed by the ICU (23.8%). The significant rise in thoracic trauma and ICU admissions among traffic accident victims may reflect an increase in the severity of accidents, potentially due to damaged road infrastructure and compromised traffic enforcement. Additionally, this increase in ICU admissions could indicate an altered healthcare organization where minor injuries were managed in field hospitals, leaving tertiary centers to handle severe cases. Furthermore, post-disaster population movements and the transfer of patients to hospitals outside the disaster epicenter likely influenced the observed demographic variations in the emergency department. Such demographic and clinical shifts due to post-disaster migration have been well-documented in recent disaster medicine literature. 24

In the evaluation of occupational accidents based on employment sector, factory workers constituted the largest group in the pre-earthquake period, whereas construction workers were predominant in the post-earthquake period. In a study by Holizki et al, 25 factory workers accounted for 31.7% and construction workers for 20.2% of occupational accidents. In another study by Özkan et al, 26 60% of cases included in the study were factory workers and 23.8% were construction workers. Our findings for the pre-earthquake period are consistent with the literature. In the post-earthquake period, occupational accidents increased proportionally among construction sector workers and public employees. We believe that the increase in the construction sector is due to the high number of workers involved in debris removal, repairs, and new construction, along with unsafe working environments caused by the nature of the disaster.

Concerning injury sites in occupational accidents, extremity injuries and cranial injuries were common in both periods. In a study by Shishlov et al, 27 the most frequent trauma region in occupational accidents was the upper extremity (29%), and in a study by Erdemli et al, 28 it was also the upper extremity (45.4%). Our findings are consistent with the literature.

## Limitations

This study has several limitations. First, it utilizes a single-center retrospective design, making it susceptible to missing data. Second, the hospital where the study was conducted sustained partial damage, and significant post-disaster population displacement occurred; thus, many patients may have sought care at field hospitals or out-of-region centers, potentially leading to an underestimation of the true regional incidence. Third, the potential confounding effects of concurrent public health dynamics, such as the late phases of the COVID-19 pandemic or altered local healthcare infrastructure, cannot be entirely excluded. Lastly, given the numerous subgroup analyses conducted, the findings should be interpreted cautiously due to the inherent risk of type I errors associated with multiple comparisons.

## Conclusion

In conclusion, disaster conditions fundamentally altered the profile of medico-legal emergencies presenting to the emergency department. The post-earthquake period was characterized by a decline in custody-forensic examinations, juxtaposed with significant increase in traffic accidents, construction-related occupational injuries, and carbon monoxide exposures due to disrupted infrastructure and subsequent rebuilding efforts. Furthermore, the rise in suicide-related poisonings among individuals without prior psychiatric diagnoses underscores the severe, widespread psychological toll of the disaster. These findings emphasize the necessity for agile healthcare resource allocation, robust occupational safety measures during debris removal, and immediate, widespread psychosocial support systems in post-disaster settings.

## References

[1] Republic of Turkey Disaster and Emergency Management Presidency. Annotated Glossary of Disaster Management Terms Earthquake Department Ankara 2014(Available at :https://www.afad.gov.tr/aciklamali-afet-yonetimi-terimleri-sozlugu)

[2] Wu Z Xu J He L Psychological consequences and associated risk factors among adult survivors of the 2008 Wenchuan earthquake BMC Psychiatry 2014 14 14 126 24779914 10.1186/1471-244X-14-126 PMC4013305

[3] Gök Y Balcı Y Göçeoğlu ÜÜ Evaluation of gender differences in adults forensic cases. Turk Klin J Forensic Med Forensic Sci 2020 17 02 133 141

[4] Farooqui M Quadri S A Suriya S S Posttraumatic stress disorder: a serious post-earthquake complication Trends Psychiatry Psychother 2017 39 02 135 143 28700042 10.1590/2237-6089-2016-0029

[5] Tascı G A Özsoy F Early psychological effects of earthquake trauma and possible risk factors Cukurova Med J. 2021 46 02 488 494

[6] Turkish Medical Association. Earthquake reports. Official Website of the Turkish Medical Association. 2023. Accessed December 17, 2024

[7] Akça H Oğlakçı oğlu A Güneri K Evaluation of forensic cases presenting to the pediatric emergency department: a single-center experience Cerrahpasa Med J. 2019 43 03 75 79

[8] Carmassi C Bertelloni C A Dell'Oste V PTSD and suicidal behaviors amongst L'Aquila 2009 earthquake young survivors Front Public Health 2021 9 590753 10.3389/fpubh.2021.590753 33643987 PMC7902690

[9] Şeker B D Effects of natural disasters on migration: earthquakes and Türkiye GocDerg. 2023 10 02 173 187

[10] Akçan R Yıldırım MŞ Isak A Tümer A R The unexpected effect of Syrian civil war in Turkey: change of forensic postmortem case pattern J Forensic Leg Med 2019 66 65 69 31226501 10.1016/j.jflm.2019.06.006

[11] Kök A N Öztürk S Tunalı İ Retrospective evaluation of 959 forensic cases treated as inpatients Adli Tip Derg. 1992 8(1–4):93 98

[12] Zeren C Karakuş A Çelikel A Evaluation of forensic cases in emergency service, Mustafa Kemal University Hospital of Medical Faculty Med J Mustafa Kemal Univ. 2015 2 07 120 130

[13] Turla A Aydin B Sataloğlu N [Mistakes and omissions in judicial reports prepared in emergency services] Ulus Travma Acil Cerrahi Derg 2009 15 02 180 184 19353323

[14] Yavuz E Turgut K Retrospective evaluation of patients who ingested drugs to commit suicide J Clin Toxicol 2021 15 03 175 182

[15] Goldney R D A global view of suicidal behaviour Emerg Med (Fremantle) 2002 14 01 24 34 11993832 10.1046/j.1442-2026.2002.00282.x

[16] Song Y Rhee S J Lee H Kim M J Shin D Ahn Y M Comparison of suicide risk by mental illness: a retrospective review of 14-year electronic medical records J Korean Med Sci 2020Dec 7;35 47 e402 33289369 10.3346/jkms.2020.35.e402 PMC7721561

[17] Goldney R D Suicide prevention is possible: a review of recent studies Arch Suicide Res 1998 4 01 329 339

[18] Ahmad S Feder A Lee E J Earthquake impact in a remote South Asian population: psychosocial factors and posttraumatic symptoms J Trauma Stress 2010 23 03 408 412 20564375 10.1002/jts.20535

[19] Aydeniz E Ünaldı M Güneysel Ö The retrospective evaluation of injuries owing to traffic collisions in the emergency department J Kartal 2014 25 01 5 12

[20] Aktaş E Ö Koçak A Zeyfeoğlu Y Solak İ Aksu H Characteristics of cases admitted to the Emergency Department of Ege University Faculty of Medicine due to traffic accidents Ann Forensic Med Meetings 2002 16 19

[21] Küçüker H Aksu A Evaluation of traffic accident cases admitted to the Emergency Department of the Fırat UniversityHospital in 1997–2001 Turk J Emerg Med 2003 3 02 11 15

[22] Varol O Eren ŞH Oğuztürk H Investigation of the patients who admitted after traffic accident to the emergency department CU Tip Fak Derg 2006 28 02 55 60

[23] Aktaş N Gülacti U Lok U Aydin İ Borta T Celik M Characteristics of the traumatic forensic cases admitted to emergency department and errors in the forensic report writing Bull Emerg Trauma 2018 6 01 64 70 29379812 10.29252/beat-060110 PMC5787366

[24] Özdemir S Özkan A Altunok İ Kokulu K Körelçiner H S Earthquake-related admissions to a trauma center located 1,100 kilometers from the epicenter: Kahramanmaras (2023) Ibnosina J Med Biomed Sci 2026 18 58 64

[25] Holizki T McDonald R Foster V Guzmicky M Causes of work-related injuries among young workers in British Columbia Am J Ind Med 2008 51 05 357 363 18302139 10.1002/ajim.20555

[26] Özkan S Kiliç S Durukan P Occupational injuries admitted to the emergency department Ulus Travma Acil Cerrahi Derg 2010 16 03 241 247 20517751

[27] Shishlov K S Schoenfisch A L Myers D J Lipscomb H J Non-fatal construction industry fall-related injuries treated in US emergency departments, 1998-2005 Am J Ind Med 2011 54 02 128 135 20635372 10.1002/ajim.20880

[28] Erdemli H Kavalcı C Suveren Analysis of work-related injuries admitted to the emergency department J Surg Arts. 2017 12 04 85 92

